# Kinase–phosphatase balance in exercise adaptation: phosphorylation programs, PTM crosstalk, and actionable gaps

**DOI:** 10.3389/fspor.2025.1743233

**Published:** 2025-12-10

**Authors:** Heming Chen, Zhihui Li, Yanyan Liu, Ying Ji, Junjie Liu, Mi Zheng

**Affiliations:** 1 Graduate School, Harbin Sport University, Harbin, Heilongjiang, China; 2 College of Science and Technology, China Three Gorges University, Yichang, China

**Keywords:** kinase–phosphatase balance, AMPK–Akt–mTOR signaling, MAPK phosphatases(MKPs), PHLPP phosphatases, phosphoproteomics, histidine phosphorylation

## Abstract

Phosphorylation is set by the opposing activities of kinases and phosphatases and this regulation likely contributes to exercise-induced adaptation. It does so by regulating mitochondrial biogenesis, muscle remodeling, and metabolic flexibility. The process by which exercise activates the AMPK, MAPK, and Akt-mTOR pathways, and how phosphatases (MKP, PHLPP, and PHPT1/LHPP) limit signal amplitude and duration to avoid maladaptive behavior, has been extensively studied. Some data suggest PHLPP2 may increase after HIIT, which could contribute to limiting Akt activity. In contrast, endurance training has been associated in some studies with relatively lower PHLPP activity; this observation may be consistent with sustained Akt-dependent mitochondrial adaptations, but direct causal evidence is limited. Systems-level phosphoproteomics unveils tissue- and time-resolved, modality-dependent phosphorylation programs and situates this axis within broader PTM crosstalk (lactylation). We outline manageable gaps linking kinase-phosphatase interactions to chromatin regulation, delineate non-canonical histidine phosphorylation, and present a condensed roadmap (time-resolving, compartment-aware phosphoproteomics integrated with epigenomic profiling) that connects enzyme function to phenotype and provides precise exercise recommendations and metabolic disease therapies.

## 1 Introduction

PTMs offer fast, reversible control of protein function and signaling during exercise, matching cell metabolism and structure to changing mechanical and energetic demands (1, 2). Distinct training modes leave unique PTM signatures: Endurance training mainly reshapes mitochondrial acetylation to promote oxidative metabolism, while resistance training primarily modifies contractile and cytoskeletal protein phosphorylation to follow strength changes (3, 4). Outside of skeletal muscle, long-term exercise induces cardioprotective remodeling, potentially through mechanisms involving titin mechanics and PTM regulation, and emerging evidence indicates metabolite-driven lactylation to be cardioprotective (5, 6).

At the signaling nexus, AMP-activated protein kinase (AMPK) demonstrates multi-post-translational modification (PTM) integration; its activity, localization, and stability are regulated by phosphorylation, acetylation, ubiquitination, and methylation, thereby connecting energetic stress to adaptive responses (7). Mechanotransduction also interacts with chemical regulation; the deformation of sarcomeric and focal adhesion proteins alters kinase/phosphatase accessibility and substrate conformation (8). Recent progress in mass-spectrometry proteomics facilitates the creation of system-level maps of exercise-regulated PTM landscapes and enables the shift from descriptive catalogs to pathway-level inference (9, 10). In this expansive domain, phosphorylation is the most thoroughly quantified and mechanistically accessible axis for linking acute exercise stimuli to synchronized metabolic and structural results. We propose that exercise adaptation is governed by kinase-phosphatase feedback circuits that establish a dynamic phosphorylation setpoint. This setpoint, which is established by the balance of kinase-driven signal amplification and phosphatase-mediated feedback, encodes the nature of the exercise stimulus and directs the specificity of long-term adaptive responses (2, 11–17).

## 2 From global PTM landscapes to phosphorylation dynamics in exercise

Exercise perturbs cellular energy homeostasis, calcium handling, redox state, and cytoskeletal tension; all these inputs contribute to the activation of kinases with opposing phosphatase activity. These factors together influence the magnitude, duration, and localization of phosphorylation signals. This framework contains AMPK that incorporates energetic stress to increase glucose uptake, fatty-acid oxidation, and mitochondrial biogenesis, while mechanosensitive scaffold structures such as titin and focal-adhesion complex control kinase access and substrate conformations (5, 7, 8). Recent phosphoproteomic studies reveal a coordinated, mode- and intensity-dependent phosphorylation program in both humans and animal models, enabling inference of upstream kinases via kinase–substrate enrichment analysis (KSEA) and HIIT workload–based analyses allow inference of potential regulatory drivers and feedback loops (3, 9–11). These individual findings together point towards a more focused investigation of the kinase-phosphatase axis as an organizing principle for dynamic control of exercise adaptation.

## 3 The kinase–phosphatase axis governing phosphorylation

### 3.1 Architecture and core control points

Protein phosphorylation, a type of PTM, is highly important in cellular signaling and function, in particular in exercise physiology, where fast and dynamic changes are essential (12). Protein kinases and phosphatases constitute a finely tuned regulatory network that determines whether substrate proteins are phosphorylated (phosphorylation), which influences subsequent downstream signaling events that are very important for how we adapt when exercising (13). Kinases add phosphate groups to specific amino acids such as serine, threonine, tyrosine, and histidine, and phosphatase remove them, which ensures that phosphorylation is dynamic and reversible (14).

**Figure 1:**
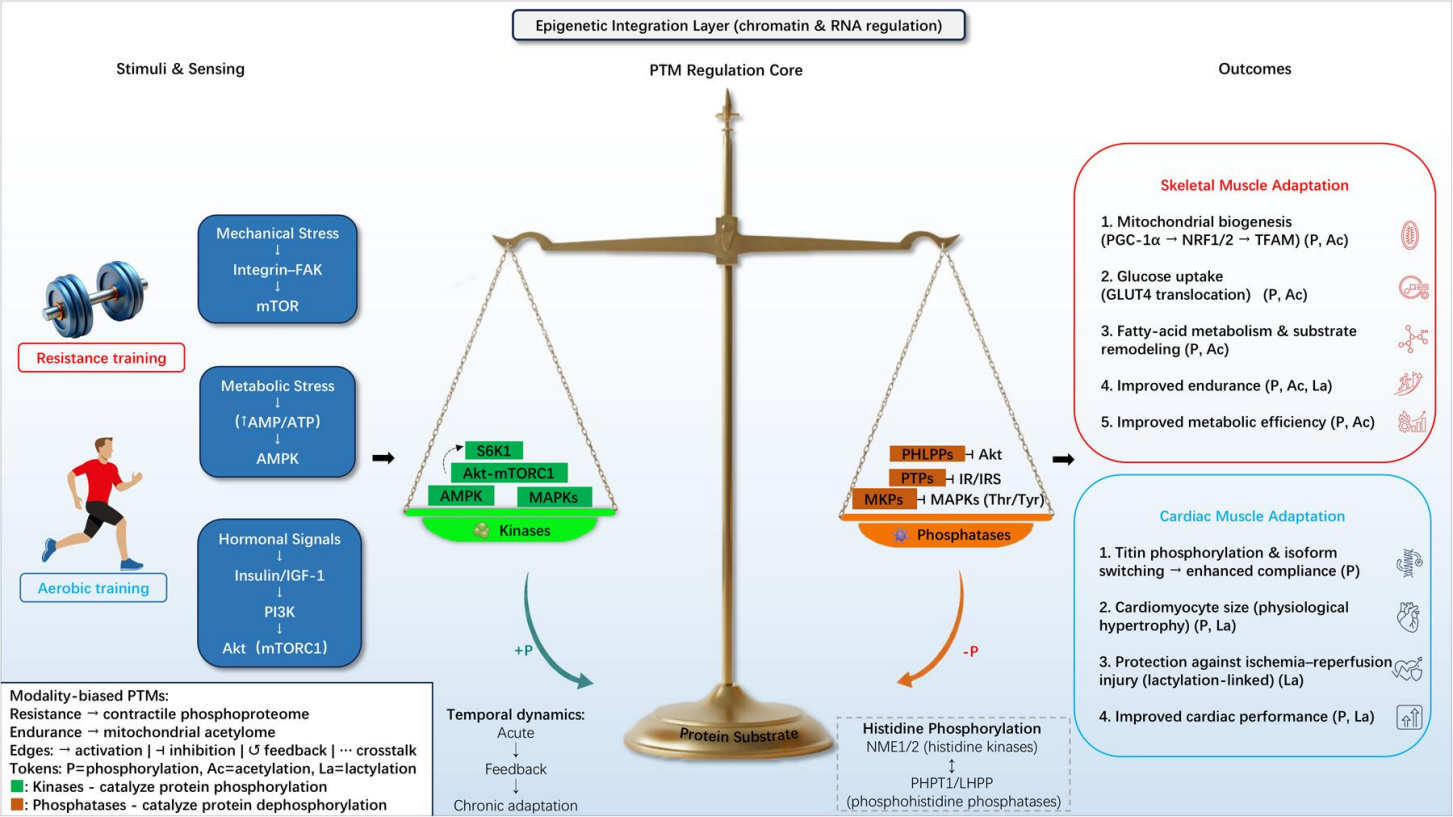
Kinase-phosphatase networks mediate PTM-dependent muscle adaptation to exercise (5, 7, 12, 16). Concise Figure Legend: Schematic of kinase (AMPK, Akt-mTORC1, MAPKs) and phosphatase (MKPs, PTPs, PHLPPs) networks regulating PTM (phosphorylation/acetylation/lactylation) dynamics in skeletal (top) and cardiac (bottom) muscle during exercise. Left: Exercise stressors (mechanical/metabolic/hormonal signals); Central: Kinase-phosphatase PTM regulation (acute vs. chronic); Right: Functional outcomes (mitochondrial biogenesis, glucose uptake).

This balance is crucial for cellular homeostasis and for coordinating signaling mechanisms activated by various mechanical, metabolic, and hormonal stressors during exercise. For example, the mitogen-activated protein kinase (MAPK) pathway, a central signaling pathway responsible for translating extracellular stimuli into intracellular responses, is tightly regulated by MAPK phosphatases (MKPs) (15). MKPs dephosphorylate MAPKs and can reduce sustained MAPK signaling; this action has been proposed to limit overactivation that may otherwise contribute to maladaptive responses (12). Additionally, histidine phosphorylation, a less explored but increasingly recognized PTM, is dynamically regulated by histidine kinases (NME1, NME2) and phosphohistidine phosphatases (PHPT1, LHPP), which have been shown to regulate cellular functions, and their possible roles in exercise-induced adaptations are emerging but not yet fully defined (16). Figure 1 provides a schematic overview of how these kinase-phosphatase networks coordinate PTM-dependent adaptations across skeletal and cardiac muscle in response to exercise stressors.

The dynamic balance maintained by kinases and phosphatases enables skeletal muscle cells to quickly adjust protein activity, gene expression, and metabolic pathways in response to acute exercise stimuli (17). This regulation promotes vital processes such as mitochondrial biogenesis, muscle hypertrophy, and metabolic remodeling (18). Disruption of this balance impairs signal transduction and hinders exercise adaptation, underscoring the critical role of kinase-phosphatase interactions in exercise physiology (13). Recent advances in the development of pHis-stable analogs, including chemically modified phosphohistidine mimetics that resist thermal and acid-mediated hydrolysis, have enabled the generation of conformation-specific antibodies and improved peptide enrichment strategies (19–21). These tools circumvent the intrinsic lability of the phosphoramidate bond and are beginning to open the field for quantitative profiling of pHis sites in physiologically relevant conditions (14, 19, 22). Complementary to these chemical tools, specialized mass-spectrometry approaches, such as neutral-loss-triggered MS/MS acquisition, high-pH sample handling (23), and Fe-IMAC variants tailored for acid-labile PTMs (22), now permit more reliable detection of pHis-containing peptides (23, 24). Coupled with improvements in chromatographic stabilization, these techniques increase the feasibility of mapping pHis dynamics under exercise stimuli (14). Given that exercise elicits acute and transient shifts in metabolic flux, redox state, and allosteric enzyme regulation, these methodological advances are particularly valuable for capturing rapid pHis cycling.

### 3.2 Stimulus encoding: kinase amplification vs. phosphatase feedback

In exercise, enhanced energetic and mechanical stimuli activate diverse kinases, thereby amplifying intracellular signaling pathways required for adaptation (17). Aerobic exercise enhances the activity of kinases like Akt and mTOR, which promote mitochondrial biogenesis and protein synthesis, respectively, thus enhancing endurance and muscle growth (17, 25, 26). Activated kinases phosphorylate critical substrates, thereby amplifying signal transduction in paths that bring about these beneficial changes. But in order for this to stop overactivating and keep cells in balance, phosphatase gives important negative feedback by dephosphorylating these kinases, which reduces kinase signaling again (13). The role played by phosphatases like MKPs is well-known in this respect -by means of MKPs, MAPKs can be deactivated, so insulin signaling, glucose homeostasis, and inflammatory responses will be affected -all of which relate to exercise-induced metabolic adaptation (12). Moreover, certain phosphatases like phosphatase 5 can also regulate cardiac adaptation to aerobic exercise through regulation of Akt/mTOR signaling and mitochondrial biogenesis; MKP-5 deficiency has been associated with enhanced endurance capacity in some models, possibly related to prolonged kinase activity, but mechanistic generalization requires further study (25). Kinase-phosphatase interplay makes sure that the strength and duration of signaling are correctly regulated to ensure that appropriate adaptive responses to exercise occur without deleterious consequences such as oxidative stress or metabolic dysregulation (12). Exercise-induced kinases as well as phosphatases give negative feedback; this is a dynamical regulatory mechanism that finely adjusts muscle's adaptation to exercise.

### 3.3 Pathway case studies and metabolic consequences

Kinases and phosphatases make phosphorylation dynamics by intersecting paths that end up controlling metabolic adjustment to exercise (27). In skeletal muscle, energy-sensing kinases, such as AMPK, facilitate glucose uptake and fatty acid oxidation while promoting mitochondrial biogenesis to enhance metabolic flexibility during the exercise-recovery cycle (28, 29). Counterregulation by phosphatases, including PTPs and MKPs, limits the amplitude and duration of signaling at crucial sites in insulin and inflammatory pathways, preventing overshoot and maladaptation (30).

PTP1B was examined as a case study with contextual variability. In models of short-term strength training and acute swimming, a decrease in hepatic PTP1B content, or a diminished association with IR/IRS, is associated with increased tyrosine phosphorylation of insulin-signaling intermediates and enhanced glucose homeostasis (3, 30–32). These findings are directionally consistent yet model-constrained: (i) most readouts are hepatic rather than myocellular; (ii) training status, age, and adiposity vary across studies; and (iii) measurements are typically endpoint rather than time-resolved, which obscures transient dephosphorylation events during recovery. Thus, while the available evidence from these models favors an inhibitory role for PTP1B on insulin signaling in low-to-moderate intensity contexts, direct data for high-intensity interval training (HIIT) are lacking; any claim that PTP1B supports “metabolic resilience” at very high intensities remains hypothesis-level and should be framed as a testable prediction rather than an established mechanism (12, 30). Priority experiments include tissue-resolved phosphatase activity profiling during HIIT, substrate-specific interactomics, and parallel assessment of inflammatory mediators that modulate PTP1B.

MKP-centered control of MAPK programs with potential trade-offs. MAPK phosphorylation orchestrates transcriptional programs balancing anabolic and catabolic processes; MKPs enforce timely deactivation to avoid signal persistence (12, 30). Notably, MKP-5 deficiency has been associated with improved endurance capacity, possibly related to prolonged kinase activity; however, whether this translates across sex, age, cardiac phenotypes, or chronic loading remains unresolved (25). Given family-level redundancy and compensatory feedback among MKPs, isoform-specific conclusions should be tempered pending multiplex perturbations and cardiac/muscle tissue-specific readouts (33–35).

Membrane signaling and non-canonical play a significant role in this process. Phosphoinositide turnover, controlled by lipid kinases and phosphatases, integrates membrane excitability, vesicular trafficking, and nutrient transport during contraction and recovery (36, 37). Beyond Ser/Thr/Tyr phosphorylation, histidine phosphorylation emerges as an additional regulatory layer influencing metabolic enzymes and adaptor proteins; yet most current evidence derives from oncology/immune contexts, underscoring the need for exercise-specific mapping of histidine kinases/phosphatases and their substrates (16, 38). To systematically summarize the core regulatory axes linking kinase-phosphatase balance to exercise-induced metabolic adaptation, Table 1 consolidates key molecules, their key targets, context-dependent mechanisms, and associated exercise scenarios, with references aligned to the preceding discussion.

**Table 1:** Key kinases/phosphatases regulating exercise-induced metabolic adaptation via post-translational modifications (PTMs).

Regulatory molecule	Key target substrate/pathway	Core regulatory mechanism	Exercise scenario specificity	References
Kinases				
AMPK	Glucose transporter 4 (GLUT4), Acetyl-CoA carboxylase (ACC)	Phosphorylates substrates to enhance glucose uptake, fatty acid oxidation, and mitochondrial biogenesis	Low-to-high intensity endurance exercise; acute energy stress	(7, 28, 39)
Akt	mTOR, FoxO1	Activates mitochondrial biogenesis; inhibits skeletal muscle protein degradation	Aerobic exercise; post-exercise recovery	(25, 40)
mTOR	p70S6K, 4E-BP1	Promotes skeletal muscle protein synthesis to support hypertrophy	Resistance training; high-intensity interval training (HIIT)	(25, 41)
MAPK (ERK1/2)	Transcription factors (c-Fos)	Orchestrates transcriptional programs balancing anabolic and catabolic processes	Acute exercise stress (aerobic or resistance training)	(12, 25)
Histidine kinases (NME1/2)	Metabolic enzymes (hexokinase)	Modulates enzyme activity via histidine phosphorylation (non-canonical PTM)	Emerging; not fully defined in exercise (inferred from non-exercise models primarily)	(16, 38)
Phosphatases				
PTP1B	Insulin receptor (IR), Insulin receptor substrate (IRS)	Dephosphorylates IR/IRS to inhibit insulin signaling; potential context-dependent role (hypothetical)	Low-to-moderate intensity exercise (hepatic focus); HIIT (unclear, myocellular data lacking)	(30–32, 42)
MKPs (MKP-5)	MAPK (ERK1/2, p38), Akt	Dephosphorylates target kinases to terminate signaling; regulates cardiac/ muscle endurance adaptation	Aerobic exercise; chronic endurance training	(12, 25)
PHPT1/LHPP	Phosphohistidine-containing proteins	Removes phosphate groups from histidine residues to reverse non-canonical phosphorylation	Emerging; exercise-specific substrates remain unknown	(16, 43)
Lipid phosphatases (PTEN)	Phosphoinositides (PIP3)	Regulates phosphoinositide turnover to modulate membrane signaling and vesicular trafficking	Muscle contraction; post-exercise nutrient transport	(36, 44–46)

The table underscores unresolved inquiries that necessitate further exploration: specifically, the myocellular function of PTP1B in high-intensity interval training (HIIT) remains undefined, and the substrate specificity of histidine kinases and phosphatases in exercise contexts has yet to be delineated (16, 30, 32). The knowledge gaps correspond with the methodological challenges previously addressed, including the constraints of static phosphoproteomic techniques. They highlight the necessity for time-resolved, tissue-specific assays to elucidate the dynamic interactions of kinase-phosphatase networks during exercise adaptation (47, 48).

Methodological caveats and directions. A good many datasets have static phosphoproteomics snapshots, which tend to underestimate site occupancy/stoichiometry and rarely measure crosstalk with acetylation or lactylation. Network -inference (kinase-substrate enrichment) can be strong but isn't causal; it is validated and necessary. More future works can apply time-resolved phosphoproteomics with compartment-aware phosphoproteomics with parallel PTM layers and proximity labeling to delineate phosphatase micro-domains and chemoproteomics aimed at substrate discovery (47, 48). They all work together to establish a regulatory framework in which the balance between kinase and phosphatase activity governs metabolic reprogramming. This makes it easier to use oxygen and handle sugar, as well as change the way things are done. However, there are still big questions about the spatiotemporal dynamics (magnitude, localization and timing) of regulatory activity.

Importantly, the regulatory logic of histidine phosphorylation in oncology and immunology may not directly translate to exercise physiology (16). Tumor and immune cells operate under chronic proliferative or inflammatory pressure, characterized by sustained glycolytic flux, altered pH homeostasis, and persistent activation of NME1/2-linked stress pathways (49). By contrast, skeletal muscle during exercise experiences oscillatory energy states, rapid ATP turnover, mechanical deformation, and tightly timed recovery phases (50, 51). These physiological constraints suggest that pHis signaling in muscle may follow different temporal dynamics, substrate preferences, and phosphatase sensitivities than those described in disease models (16, 49, 52). Additionally, the pH microenvironment of contracting muscle fluctuates more dramatically than in oncogenic settings, which is relevant because the phosphoramidate bond is acid-labile (53); this may impose distinct stabilization requirements for pHis-containing enzymes during exercise. Mechanical load, Ca²^+^ microdomain signaling, and mitochondrial respiratory shifts further introduce regulatory layers that are absent in tumor or immune niches (53). Thus, enzymes such as hexokinase, GAPDH, or mitochondrial carrier proteins, which undergo pHis modification in cancer metabolism, may respond differently under workloads where substrate availability, redox balance, and AMP/ATP ratios fluctuate within seconds (54).

## 4 Mechanistic insights, knowledge gaps, and methodological roadmap

Protein phosphorylation, the primary post-translational modification (PTM) that directs exercise-induced adaptations, is tightly regulated by the coordinated action of kinases and phosphatases (27, 55). This process also serves as a vital link for connecting energy stress signals, mechanical cue transmission, and interplays between various PTMs (9, 56). AMPK, mTOR, MAPK, and other key kinases are stimulated by exercise stimuli: AMPK mediates glucose uptake via phosphorylation of glucose transporter 4 (GLUT4) and fatty acid oxidation via phosphorylation of acetyl-coenzyme A carboxylase (ACC), sustaining energy homeostasis during exercise (7, 28); mTOR activates p70 ribosomal S6 kinase (p70S6K) and eukaryotic translation initiation factor 4E-binding protein 1 (4E-BP1) in skeletal muscle for protein synthesis needed in resistance training hypertrophy (25, 57); and the MAPK family, such as the ERK1/2 family, coordinates anabolic and catabolic transcriptional programs through regulating transcription factors like c-Fos (12, 58).

Meanwhile, phosphatases build up an essential negative feedback circuit: MAPK phosphatases MKPs inactivate MAPKs by dephosphorylation and inhibit excessive MAPK activation of inflammatory response (12); the PHLPP family inhibits Akt activity to prevent cell damage caused by sustained signal transmission (25); and P-type phosphatases PHPT1 and LHPP reverse the modification of histidine phosphorylation and keep the reversibility of non-canonical PTMs (16). These three groups of phosphatases ensure that the intensity and duration of exercise signals match physiological demands.

Different kinds of exercises have different ways to control these kinases and phosphatases; this is important for how they adapt to the exercise (59). Acute energetic stress from HIIT causes PHLPP2 expression. One important role is as a suppressor of Akt overactivity, which has been proposed to serve a protective role against an unhealthily high proliferation of muscle cells during metabolic stress (6); endurance training, on the other hand, keeps PHLPP activity low, which creates an environment that allows for the sustained Akt signaling needed for mitochondrial biogenesis. This effect is directly linked to the activation of oxidative metabolism genes by peroxisome proliferator-activated receptor *γ* coactivator 1α (PGC-1α) (7, 60). Notably, several research gaps remain: the expression differences of PHLPP1 and PHLPP2 between type I (oxidative) and type II (glycolytic) muscle fibers, as well as the impact of their subcellular localization on signal efficiency, have not been clarified (25); although histidine phosphorylation is known to regulate the activity of metabolic enzymes such as hexokinase (16, 61), the substrate profiles of its associated kinases (NME1/2) and phosphatases in exercise contexts still rely on indirect evidence from oncology and immunology fields (38). To directly address the knowledge gap regarding histidine phosphorylation, future work should incorporate acid-free sample preparation pipelines, rapid tissue freezing during *in vivo* contractions, and compartment-resolved isolation of mitochondria and myofibrils (53). These approaches are essential for preserving pHis stability during the transient metabolic transitions of acute exercise. Time-resolved phosphoproteomics specifically optimized for pHis detection would allow measurement of rapid histidine dephosphorylation events that may occur within seconds of contractile onset or metabolic recovery—events that are invisible to conventional workflows (16, 53). Furthermore, integrating pHis-sensitive proteomics with metabolomics and isotope-tracing (fluxomics) could reveal how histidine phosphorylation unites metabolic switches—such as glycolytic burst, PCr depletion, or mitochondrial engagement—to enzyme activity during exercise. Consequently, it is essential to perform exercise-oriented studies employing mass spectrometry for the examination of Ser/Thr/Tyr phosphorylation (9, 10), to address this knowledge deficiency (53).

Methodological limitations restrict the depth of mechanistic analysis. Static phosphoproteomics cannot capture transient dephosphorylation events of protein tyrosine phosphatase 1B (PTP1B) during HIIT (47, 62), necessitating the combination of time-resolved techniques and tissue-specific sample preparation (laser capture microdissection) to analyze phosphatase activity dynamics; chemoproteomics can identify novel substrates of phosphatases such as MKP-5 by capturing phosphatase-substrate complexes via activity-based probes (48, 63); and proximity labeling techniques (BioID) can clarify the localization of phosphatases in microdomains such as sarcomeres and mitochondria (63), explaining the spatial regulatory mechanisms underlying their substrate specificity (25). The combination of such technologies will move exercise phosphatase studies from “descriptive profiling” to “causal mechanistic” research. To address these limitations, we described the important methodological directions for combining time-resolved phosphoproteomics with spatially resolved and epigenomic technologies (Table 2).

**Table 2:** Multi-omics integration pipeline.

Research objective	Method/technology	Mechanistic role/data output	Spatial/temporal resolution	Key advantages and potential limitations	References
Capture exercise-induced dynamic protein phosphorylation events	Time-resolved phosphoproteomics (MS-based)	Measures changes in Ser/Thr/Tyr phosphorylation levels over the exercise time course	High temporal resolution (minute scale)	Tracks transient signaling events; interprets kinase/phosphatase activity dynamics. Requires large sample numbers; complex experimental design	(64)
Define tissue- or cell-type–specific PTMs	Laser Capture Microdissection (LCM) + MS	Profiles phosphorylation in specific muscle fiber types or microregions	High spatial resolution (single-cell or sub-tissue level)	Identifies fiber-type or subregional differences. Technically demanding; sample loss during preparation	(65, 66)
Determine phosphatase substrates and activity spectrum	Chemoproteomics (activity-based probes)	Captures phosphatase–substrate complexes and identifies novel substrates	Can incorporate temporal or tissue-specific sampling	Directly identifies functional substrates; enhances causal mechanistic interpretation. Requires specialized probes; may miss low-abundance substrates	(67, 68)
Reveal subcellular localization and microdomain regulation	Proximity labeling (BioID/TurboID)	Maps phosphatase localization within cellular microdomains (mitochondria, sarcomeres, etc.)	High spatial resolution (subcellular level)	Explains spatial mechanisms underlying substrate specificity. Requires engineered fusion proteins; limited applicability in *in vivo* systems	(69–71)
Cross-layer mechanistic integration	Epigenomic analyses (ChIP-seq/ATAC-seq/CUT&RUN)	Reveals how PTM signaling shapes chromatin accessibility and gene expression	Compatible with time-series sampling	Links signaling events to transcriptional responses, supporting causal mechanistic chains. Data integration is complex; requires multi-layer bioinformatics	(72–74)

This table summarizes key methodological strategies for dissecting kinase–phosphatase signaling during exercise adaptation and highlights persistent gaps that limit mechanistic resolution. Despite advances in time-resolved phosphoproteomics and chemoproteomic substrate mapping, spatial heterogeneity across muscle fiber types and subcellular microdomains remains insufficiently characterized. Likewise, integrating phosphosignaling with chromatin accessibility and transcriptional outputs is hindered by the limited temporal resolution of current epigenomic assays. Generally speaking, these constraints underscore the need for spatiotemporally resolved, multi-omics frameworks to define how localized signaling events are converted into coordinated adaptive responses.

## 5 Conclusions and perspectives

Phosphorylation, regulated by kinases and phosphatases, is a key molecular axis implicated in exercise-induced adaptations. It controls important cellular processes like mitochondrial biogenesis, muscle hypertrophy, and metabolic flexibility. This balance is not merely a simple on-off switch; rather, it constitutes a complex regulatory hub that uses mechanical, metabolic, and hormonal signals to make sure that molecular responses match the needs of exercise. HIIT may induce an increase in PHLPP2 expression, which has been proposed to help restrain excessive Akt activation and prevent maladaptation, whereas during endurance training, PHLPP activity tends to remain low, supporting Akt-dependent mitochondrial biogenesis.

We currently lack essential knowledge about the functional interaction between phosphorylation dynamics and epigenetic regulation. Exercise is accompanied by histone acetylation, such as heightened acetylation at mitochondrial-gene promoters during endurance exercise, and can also trigger DNA methylation at loci linked to fiber type during resistance training. However, the mechanisms by which the kinase–phosphatase network facilitates these epigenetic modifications are not yet fully understood. One important question is whether AMPK, the main energy-sensing kinase, connects energetic stress to chromatin remodeling by adding phosphate groups to histone-modifying enzymes like acetyltransferases or deacetylases, and another key question is whether the regulation of Akt signaling by PHLPP alters the activity of methyltransferases like EZH2. These enzymes are responsible for creating the epigenetic memory of muscle fiber type. Furthermore, the function of non-canonical phosphorylation, particularly histidine phosphorylation, in exercise-induced epigenetic regulation is inadequately investigated and constitutes a significant area for future research.

Future work should integrate time-resolved phosphoproteomics with epigenomic profiling—such as ChIP-seq for histone marks and ATAC-seq for chromatin accessibility—to build causal paths from kinase–phosphatase activity to epigenetic modifications, gene expression, and ultimately physiological adaptation. The advancement of high-resolution quantitative phosphoproteomics and single-cell phosphoproteomics will be crucial for resolving dynamic kinase and phosphatase activities across fiber types and cellular subpopulations. Moreover, CRISPR-Cas9–mediated conditional knockouts and chemogenetic/optogenetic tools will help establish causal links between enzyme activity and adaptive phenotypes, moving beyond inferences from correlative networks.

Finally, fixing methodological inconsistencies is important for making the results clearer. For example, most studies on PTP1B and exercise focus on liver tissue, which doesn't really explain how it works in myocyte insulin signaling. To address these discrepancies, supplementary tissue- and cell-specific models are essential. Because phosphatase families have some overlap, like how MKP isoforms regulate MAPKs, we need to make several changes to stop mistakenly linking phenotypes to one enzyme (75, 76). Integrated mechanistic insights could inform translational strategies with potential applications in metabolic disease treatment, athletic performance, and sarcopenia mitigation—though substantial translational work will be required. These advances have the potential to shift aspects of exercise physiology towards more mechanistic, molecularly specific frameworks.
